# Reframing professional identity through navigating tensions during residency: A qualitative study

**DOI:** 10.1007/s40037-022-00709-9

**Published:** 2022-03-17

**Authors:** Wil L. Santivasi, Hannah C. Nordhues, Frederic W. Hafferty, Brianna E. Vaa Stelling, John T. Ratelle, Thomas J. Beckman, Adam P. Sawatsky

**Affiliations:** 1 Center for Palliative Care, Duke University School of Medicine, Durham, NC USA; 2 Division of General Internal Medicine, Mayo Clinic, Rochester, MN USA; 3 Program in Professionalism and Values, Mayo Clinic, Rochester, MN USA; 4 Division of Community Internal Medicine, Mayo Clinic, Rochester, MN USA; 5 Division of Hospital Internal Medicine, Mayo Clinic, Rochester, MN USA

**Keywords:** Professional identity formation, Social cognitive theory, Postgraduate medical education, Qualitative research

## Abstract

**Introduction:**

Professional identity formation (PIF) is the internalization of characteristics, values, and norms of the medical profession. An individual’s identity formation has both psychological and sociological influences. Social psychology may be useful to explore the interactions between the psychological and sociological aspects of PIF. In this study, we explored how resident physicians navigated tensions between professional ideals and the reality of medical practice to characterize PIF during residency training.

**Methods:**

Using constructivist grounded theory, the authors conducted 23 semi-structured interviews with internal medicine residents. Interview transcripts were processed through open coding and analytic memo writing. During data gathering and analysis, the authors utilized Social Cognitive Theory, specifically the bidirectional influence between person, behavior, and context, to analyze relationships among themes. Theoretical insights were refined through group discussion and constant comparison with newly collected data.

**Results:**

Residents described tensions experienced during residency between pre-existing ideals of “*a good doctor*” and the realities of medical practice, often challenging residents to reframe their ideals. The authors provide evidence for the presence of dynamic, bidirectional influences between identity (person), behavior, and environment (context), and demonstrate how PIF is informed by a complex interplay between these elements. The authors present two examples to demonstrate how residents reframed their ideals during residency training.

**Discussion:**

The complex bidirectional influences between person, behavior, and context, informed by SCT, helps illuminate the process of PIF in residency training. This study highlights the effects of the context of residency training on the development of residents’ professional identities.

**Supplementary Information:**

The online version of this article (10.1007/s40037-022-00709-9) contains supplementary material, which is available to authorized users.

## Introduction

Professional identity is “a representation of self, achieved in stages over time during which the characteristics, values, and norms of the medical profession are internalized, resulting in an individual thinking, acting, and feeling like a physician [1].” Key features of an individual’s professional identity include what a professional believes, knows, and does [2]. Professional identity *formation* (PIF) is “an adaptive developmental process that happens simultaneously at two levels: (a) at the level of the individual, which involves the psychological development of the person and (b) at the collective level, which involves the socialization of the person into appropriate roles and forms of participation in the community’s work [1, 3].”

While PIF is defined as *both* an individual and a social process, most medical education literature has focused separately on either the psychological or sociological facets of identity formation. At the psychological level, PIF is an individual process of incorporating experiences into an evolving concept of self [4, 5]. Psychological studies of PIF use theories from developmental psychology (e.g. Kegan’s model of adult development) to describe stages across a professional’s career [5, 6]. However, internal processes alone cannot capture the complexity of PIF, as context and relationships are critical [5, 7, 8]. At the sociological level, PIF is a socialization process influenced by experiences, role models, and mentors, all within the larger context of learning environments and healthcare systems [5, 8]. Sociological models highlight the complexity of contributors to PIF in medical education. While these separate lines of examination are useful for characterizing aspects of PIF, examination of the dynamic interactions between the psychological and sociological influences may provide a deeper understanding of PIF.

**Fig. 1:**
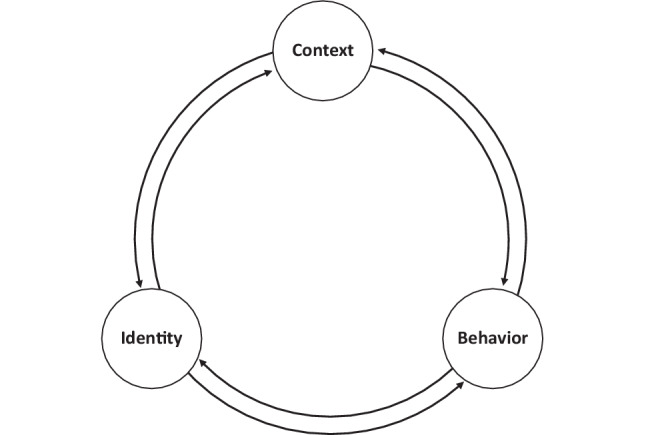
*Schematic of the bidirectional influence between identity, behavior, and context, adapted from Bandura’s Social Cognitive Theory. *Bandura’s “reciprocal determinism” described that behavior, personal factors, and environmental influences operated interactively as determinants of one another. We have adapted this model, focusing on “identity” as a summarization of “personal factors” to represent the process of professional identity formation during residency as a complex, dynamic bidirectional interaction between identity, behavior, and context

Bridging sociology and psychology, social psychology seeks to explain how social influences affect an individual’s thoughts, feelings, and behaviors. A social psychological approach to studying identity examines the dynamic interaction between individual agency and the sociological influences on identity formation [9]. Lewin, a pioneer of social psychology, proposed that human behavior is shaped by the interaction between a person and their environment [10]. Expanding on Lewin’s work, Bandura introduced Social Cognitive Theory (SCT), proposing that personal factors, environmental factors, and behaviors exert bidirectional influence on each other, a process he named “reciprocal determinism [11]” (Fig. 1). While Bandura believed that a person’s thoughts and actions are shaped by environmental influences, an individual also has agency to shape their environment through thoughts and actions [11, 12]. Bandura acknowledged that many factors may lead to a given effect, that factors contribute unequally, and that factors’ influences vary over time [11]. Bandura further describes the personal factors that affect behavior as what people “think, believe, and feel [11].” Given this definition aligns closely with our definition of identity, we have adapted the model to place identity as the personal factor and used this model to guide our data analysis. While previous frameworks outline the internal (e.g. conscious reflection) and external (e.g. mentors, role models, and the learning environment) factors that shape PIF [5], examining the bidirectional influences of factors—namely identity, behavior, and context—may provide a lens for studying the dynamic nature of PIF and integrating psychological and sociological determinants of professional identity and behavior.

Understanding these bidirectional influences may clarify identity tensions faced by trainees. For physicians, residency is a critical time for navigating tensions as residents “reconcile dissonance between the stated values of the medical profession and the realities of medicine as practiced in the real world,” including tensions between values within medicine [7, 13, 14]. Residents balance the tensions inherent to the dual role of being a physician and a trainee [15]. Residents also balance the tension between standardization of medical training and a desire for diversity and individuality within the profession [16]. These tensions often result in residents staging a performance to meet assessment expectations, potentially hindering personal and professional growth [17–19]. Expectations of residents can place undue strain (e.g. the demands of the role outstrip the resources to fulfill the role) and conflict (e.g. the role clashes with the demands of other roles), causing stress as residents navigate these tensions [20]. Exploring how residents navigate these tensions may provide insight into the processes of PIF during residency training [21].

The purpose of this study was to explore how residents navigate tensions during residency training and how that navigation affects resident PIF. Thus, we examined the experience of navigating tensions during residency through the lens of the bidirectional influences of person, behavior, and context, to further the conceptualization of PIF within medical education.

## Methods

We used a constructivist grounded theory approach, using the experience of “navigating tensions” to explore the PIF process and the theoretical framework of “reciprocal determinism” from SCT to inform our data analysis [11, 12]. We chose grounded theory because we wanted to build upon the current theoretical understanding and develop a new model that provided a new perspective of PIF in medical education. We chose a constructivist approach because it acknowledges the researcher’s involvement in the construction and interpretation of data [22]. The research team was composed of faculty members that completed training in the studied residency program in the last 5 years (WLS, HCN, BEVS, JTR), reflecting on their roles as previous resident and current faculty member. One researcher (APS) has advanced training in medical education and qualitative research, helping guide the analysis process with opportunities to reflect on both the theory and practice of medical education. One researcher (FWH) is a sociologist who has written extensively on professionalism in medical education and brings a sociological perspective to PIF in medical education [23, 24]. Throughout data collection and analysis, the team met to discuss data analysis, challenge each other’s assumptions, provide unique ideas to frame data collection and analysis, and discuss theoretical framings that informed our research.

The Mayo Clinic Institutional Review Board approved this study (IRB ID 16-010131).

### Setting and participants

Data were collected between February and May 2017. We sent email invitations to all 144 categorical residents from the Mayo Clinic Internal Medicine Residency Program in Rochester, MN to participate in one-on-one semi-structured interviews conducted over the noon hour. From those invited, we monitored our recruitment to ensure that we sampled residents from all three postgraduate classes to obtain a broad perspective on identity formation. We believe that tensions are experienced throughout residency and we sampled residents to have a broad exploration of these tensions across residency training. We conducted interviews with 23 internal medicine residents: 5 postgraduate year (PGY)-1 residents, 9 PGY‑2 residents, and 9 PGY‑3 residents. Fifteen (65%) participants were male, reflecting the gender balance of the residency program that academic year.

### Data collection

We developed an interview guide that contained questions about the meaning of a “good doctor,” tensions between residents’ ideals and the reality of medical practice during residency, navigation of those tensions, and the relationship between navigating tensions and the meaning of “*a good doctor”* (see the Appendix in the Electronic Supplementary Material). A team member (FWH) with experience in interviewing and with no leadership or evaluation role within the residency program conducted all interviews. The semi-structured format allowed the interviewer to follow-up on ideas raised by participants [25]. Data analysis occurred simultaneously with data collection, allowing the interview guide to evolve over time, informing specific areas of emphasis for subsequent interviews. Interview transcripts were de-identified prior to coding and analysis by the research team.

### Data processing

Interview transcripts were uploaded to NVivo 10 (QSR International, Australia). The entire research team read the first three transcripts and developed an initial codebook through open coding, analytic memos, and group discussion. This codebook was applied to the first five interviews and refined through an iterative process. Through the iterative process of data collection and analysis, we developed axial codes—codes that organized the open codes into broader categories. The codebook of axial codes was applied to all the interview transcripts and refined through additional discussion.

During the process of axial coding, prior to the completion of data collection, we explored theoretical frameworks that would help explain relationships between identified themes [22]. We identified SCT and “reciprocal determinism” [11, 12] as a theoretical framework that allowed us to organize and analyze the bidirectional influences among central themes, a process of “theory-informing inductive data analysis [26].” This allowed us to explore several theories to explain our findings as we collected data and to subsequently adapt our data collection given this theoretical framework. We subsequently engaged in “theoretical coding,” a process that allows researchers to code the relationships between themes [22]. Using the bidirectional influences of “reciprocal determinism,” we formed six theoretical codes, each code representing a unidirectional relationship between person, behavior, and context. We laid these codes over our inductive (i.e. open and axial) codes to facilitate data analysis and the development of theory. Two investigators (WLS, APS) applied this theoretical code structure to all 23 interviews.

### Data analysis

Using the theoretical codes and representative quotations, we identified patterns and themes addressing the process of navigating tensions and the relationship to identity formation. Data analysis was supported by analytic memo writing; the authors engaged in memo writing throughout the analysis process to capture codes, themes, and reflect on relationships between identified themes and categories. Through constant comparison between our analysis, existing theory, and subsequent data collection, we utilized reciprocal determinism as an explanatory model to frame our findings and develop a conceptual model of PIF that explains the dynamic development of professional identity through the navigation of tensions during residency [11, 12]. Through this process, we reached consensus on theoretical sufficiency, achieving a thorough understanding of the theoretical concepts.

## Results

Upon entering residency, residents had a pre-existing ideal of “*a good doctor*.” During residency, they experienced tensions between these pre-existing ideals and the reality of medical practice, challenging residents to reframe their ideals as they navigated those tensions. The process of navigating tensions was guided by reciprocal determinism—the bidirectional influences between their developing identity (person), behaviors, and the clinical learning environment. We describe the bidirectional influences we identified and provide two examples of reframed ideals situated within reciprocal determinism. Participant quotes are indicated in parentheses by participant number and residency year (e.g., 10, PGY-3).

### Relationship between identity and behaviors

During training, residents’ identity guided their behavior, and enacting those behaviors refined their identity. A second-year resident described identity as “*an integral part of yourself that influences your actions and behaviors*” (12, PGY-2). Another resident voiced his goal “*to be empathetic, to be compassionate, to show love for my patients, to be present*”; these goals were refined through action during residency: “*when you’re an intern, that’s your first chance to practice and figure out what those things mean to you in practical terms*” (17, PGY-3). The residents’ actions during training influenced their developing identities. A second-year resident reflected: “*what makes you a good doctor is what you’re doing; by doing the things that you’re doing, you’re defining yourself, and that has to be a big part of who you are*” (7, PGY-2). Residency training provided the opportunity to act out one’s identity and refine that identity through seeing the effects of those actions.

### Relationship between context and identity

Residents discussed the influence of context on their developing identities. A first-year resident recognized that “*society recognizes me as a doctor now*” (20, PGY-1). The resultant societal and professional expectations shape identity, as one resident described: “*The norms of the profession are characteristics that I want to develop; I want to be somebody who treats people with respect and listens, who is thorough, who communicates clearly and is a team player*” (6, PGY-2). Residents also discussed the influence of supervising physicians’ expectations on their identity: “*When you know that someone is expecting something of you, part of your own expectation is to meet that standard*” (2, PGY-3). Therefore, societal, cultural, and supervisor norms and expectations influence developing identity.

While context influenced identity, residents felt that they could influence their environment in limited ways to align with their developing identities. Residents chose role models and role-modeled behaviors that aligned with their desired identity: “*When I see some way that a provider explains something, or their style, or how they interact with ancillary staff, I cherry pick that and put it into my framework for what makes a good doctor. When I see something I don’t like, I note that too*” (23, PGY-2). Through choosing role models, residents can alter their context and choose how it shapes their identity.

### Relationship between context and behaviors

Residents described bidirectional influences between context and behaviors. For example, when residents were provided autonomy to make clinical decisions, they grew in responsibility for patient care. Supervisors could constrain autonomy and influence behavior: “*rather than being treated like a colleague, they have their thumb on you. They tell you what to do or there are going to be consequences*” (4, PGY-3). In certain clinical environments residents enacted roles that constrained their behavior and affected their identity. One second-year resident reported acting “*like a scribe*” (9, PGY-2). Behavior and identity were also shaped by relationships with their peers: “*The way your colleagues judge you is based on your communication with them, diagnosing, prognosing, and managing disease and issues in a good way*” (15, PGY-1).

### Reframing the ideals of a “good doctor” in residency

Given the bidirectional influence between identity, behavior, and context during residency, we provide two examples exemplifying how bidirectional influences shape residents reframing their ideals while navigating tensions. Through these examples, we demonstrate that the process of professional identity formation includes the reframing of professional ideals and occurs through the complex interplay between person, behavior, and context.

#### “*A good doctor convinces patients to accept their recommendations*” (1, PGY-3)

This third-year resident started residency thinking that an ideal doctor is “*able to make the right diagnosis [and] the right treatment plans*,” “*make [the patient] feel cared about,” and “meet the patient’s expectations*.” Ultimately, a good doctor not only makes the right decisions and recommendations, but “*convinces patients to accept their recommendations.*” However, he experienced tension through several experiences where patient expectations could not be met (e.g. “*their initial expectation was that he would be treated with more chemotherapy and cured, but that wasn’t something that I could offer*”) or where the patient rejected his recommendation. These experiences challenged his sense of identity as a “good doctor,” as he recognized “*if my satisfaction with being a doctor is contingent on my patient following everything I tell them, I will be a failure.*”

**Fig. 2:**
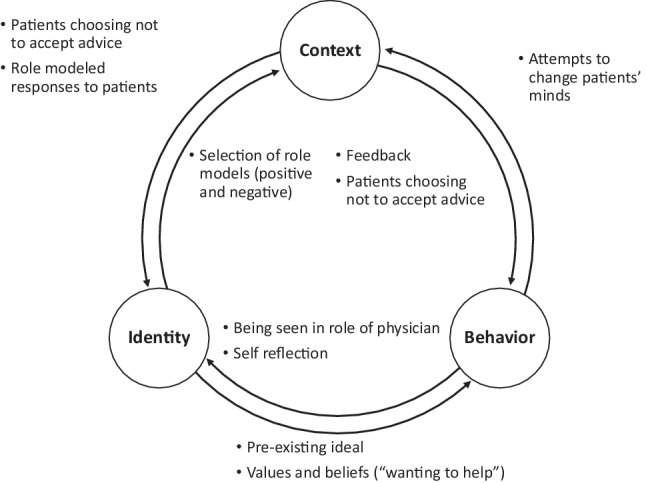
Schematic of professional identity formation as the bidirectional influences between identity, behavior, and context in reframing *“A* *good doctor convinces patients to accept [his/her] advice.”* Arrows represent directional influences between identity, behavior, and context. Associated bulleted examples were identified by the resident as factors in reframing his ideal of “*a* *good doctor convinces patients to accept [his/her] advice*” to “*a* *good doctor is a* *consultant; patients make decisions for themselves.*” These bulleted examples are associated with the adjacent arrow

Through the bidirectional influence between his developing identity, enacted behaviors, and social context, the resident reframed this initial ideal (Fig. 2). He engaged in behaviors that reflected his initial ideal, attempting to meet patient expectations and convince patients of his recommendations. When these behaviors were not met with affirming outcomes, he reflected on these patient interactions and “*talk[ed] with my co-residents, [attendings], and fellows*” to process those experiences. He selected role models to better understand how to approach the patient-doctor relationship. His clinical encounters were observed, and supervisors provided explicit feedback about how to approach shared decision-making. Through these experiences and feedback, he reframed his initial ideal to include an expectation that patients may reject his/her recommendations: “*I want them to, but my expectation is that I am consulting. I’m helping them make the best decision, and I give them recommendations. If they choose to abide by them, that’s wonderful*.”

#### “*A good doctor knows everything*” (2, PGY-3)

This third-year resident described her initial ideal that “*a good doctor*” possesses “*knowledge, first and foremost*.” At the start of training, she had the expectation: “*I’m a new doctor. I’m supposed to be knowledgeable. I’m supposed to know all these things, so I won’t have to fake it.*” However once into training, she realized “*that no one has all the knowledge and it’s not realistic to expect that. You realize that no one really knows everything, you don’t always have a perfect answer.*” This challenged her developing identity, as she continued to feel the weight of expectation: “*I’m the doctor now, I’m supposed to have answers.*”

**Fig. 3:**
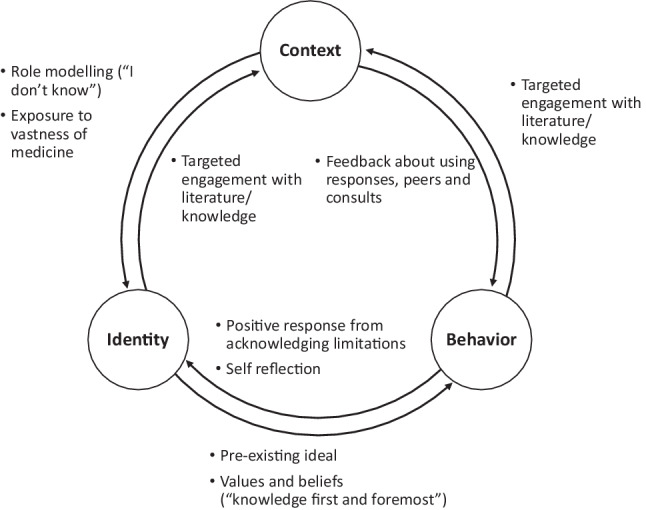
Schematic of professional identity formation as the bidirectional influences between identity, behavior, and context in reframing “*A* *good doctor knows everything.*” Arrows represent directional influences between identity, behavior, and context. Associated bulleted examples were identified by the resident as factors in reframing the ideal of “*a* *good doctor knows everything” *to *“a* *good doctor knows his/her limitations and asks for help.*” These bulleted examples are associated with the adjacent arrow

Bidirectional influences between her identity, behavior, and context illustrated the complexity of navigating this tension, leading her to reframe her ideal (Fig. 3). In trying to “*know everything,”* she attempted to demonstrate to patients and attendings the extent of her knowledge. Through her struggle, she began to realize that physicians, patients, and families did not expect perfect knowledge, and even responded well when she acknowledged her limitations. Faced with the vastness of medical knowledge, she realized it was unrealistic for one physician to “*know everything*.” This was reinforced through role modeling: “*One of the most powerful modeled behaviors is when the [attending], who is an expert, says ‘I don’t know’ or ‘I’m going to ask my colleague for help.’ It models that no one has all the answers, and that it’s not realistic*.” Through experience, feedback, and role modeling, she reframed her ideal to “*a good doctor knows her limitations and asks for help*” and that “*it is a better skill to use the resources around you than to try and know everything yourself.*”

## Discussion

In this study, residents reframed their definition of a “good doctor” during residency because of navigating tensions between their preconceived ideals of medicine and the realities of medical practice. Bidirectional influences between residents’ evolving identities, the behaviors they engaged in during residency, and the context of residency education affected the reframing of residents’ identities as “good doctors” when faced with these tensions. A resident’s developing identity guided their behavior and shaped their social context, and it evolved through their interactions within the residency training environment. SCT describes how the complex interaction of previously identified factors important to PIF, namely experiences, reflection, role models, mentors, and the learning environment, affects the development of residents’ professional identities. Growing evidence demonstrates that PIF is an individualized, non-linear process influenced by the formal, informal, and hidden curricula [27]. Exploring the bidirectional influences between identity, behavior and context can illuminate both the socialization process shaping an individual’s professional identity and an individual’s agency to shape context and relationships to align with their developing professional identity. Thus, SCT may provide a useful framework to further investigate the interaction between influences of PIF.

While residency training aims to help residents attain the knowledge, skills, and attitudes to be excellent physicians, the residency environment also presents challenges to their developing identity. Self-doubt and imposter syndrome are prevalent among resident trainees and are associated with increased anxiety, depression and burnout [28]. The culture of residency training, including constant critique and messaging that help-seeking and uncertainty are weaknesses, can perpetuate self-doubt and imposterism [28, 29]. Specific events can even lead to shame, particularly in training where residents compare themselves to others and are performance-focused, resulting in isolation, disengagement and impaired wellness [30]. In our study, we demonstrate that initial ideals may be unrealistic (e.g. “*A good doctor knows everything*”) and need to be reframed during training. While we focused on reframing ideals in a positive way, we can imagine how either failure to reframe these unrealistic ideals or framing them in less productive ways may lead to self-doubt, shame, imposterism, and their untoward effects.

In this study, role models and mentors had a significant effect on reshaping ideals. Role models are an important part of clinical training, especially as they perpetuate healthy behaviors and learners are encouraged to reflect on behaviors before adopting them [31]. To encourage modeling of healthy behaviors, there are calls to make PIF more explicit in residency training [32–34]. Structured opportunities for reflection and peer engagement may provide an opportunity for educators and peers to openly discuss important aspects of physician identity and intentionally affect PIF [35, 36]. While these initiatives have many potential benefits, including enhanced social support, stress reduction, and improved coping [35], they often occur outside of work. While this space can encourage reflection, it may lead residents to view these efforts as less valuable than the structured curriculum. Studies demonstrate that supervisor support, conversations with others, and changing the culture of assessment in medicine may help mitigate shame and self-doubt and support growth and identity development [18, 19, 30, 37]. Our study supports calls for supervising physicians to demonstrate intellectual and emotional candor to promote learning and combat the unrealistic expectations in medical training and practice [38, 39]. Understanding the challenges residents face and how they navigate those tensions can provide opportunities to discuss and reflect on those tensions within the workplace.

Attempts to measure PIF within medical education are influenced by calls to add identity (“is”) above behavior (“does”) to Miller’s Pyramid, a model for educational assessment [40–43]. Bidirectional influences between identity and behavior suggest that they are not discrete entities to be assessed separately. In addition, aspects of identity, especially attitudes and values, are private experiences, limiting reliable assessment [44]. There is likely value in multiple, varied assessments of resident behaviors and the values, attitudes, and beliefs that drive those behaviors; however, the assessment of observable behaviors and values may not fully capture professional identity [44, 45]. Given the significant contribution of context to PIF, it is also critical to assess the contextual factors within residency training that affect PIF. Programs like the ACGME’s Clinical Learning Environment Review [46] are important to examine the ways the structure of residency training shapes residents’ identities.

Our study has several limitations. First, we did not explore the origins of pre-existing ideals, only how residents’ ideals were shaped during residency training. There are widely accepted premises within medicine, similar to our study, that are inherent to the culture and implicitly transmitted through social interaction [23, 47]. Future research could utilize SCT to further examine how these premises integrate into the developing professional identities of medical trainees. Second, we did not assign direction to the developing identities, either positive or negative, but remained neutral in examining the process of navigating tensions. Additional research should continue to examine how the sociocultural context of medicine shapes professional identities, including the navigation of identity conflicts of trainees. Research examining sociocultural influences on PIF is limited; SCT can help examine ways that “learners from traditionally under-represented cultures do not fit in with the identity of the dominant health professions culture” and explore the agency of learners to reshape their environment [48]. Lastly, the specific examples presented in our results may not be transferrable to residents in other specialties with different training structures and durations, clinical experiences, and patient populations. The theoretical underpinning of bidirectional influences between context, identity, and behavior is transferrable and provides opportunities to explore examples in other contexts.

## Conclusion

Residency is a critical time for the developing physician, exposing learners to multiple tensions that exist between the ideals and the realities of medical practice. SCT provides a framework to explore PIF in residency, highlighting the bidirectional influences between identity, behavior, and context. As residents engage in clinical experiences, receive feedback, and follow role models, they reframe their ideals while navigating tensions faced during residency. Sensitivity to these processes and assessment of the context of medical education can provide educators with opportunities to facilitate residents’ intentional reflection on professional identity and recognize and mitigate negative influences on PIF.

## Supplementary Information

Interview guide for the study: “Reframing professional identity through navigating tensions during residency: A qualitative study”
