# Supplementary material for: Reframing professional identity through navigating tensions during residency: A qualitative study
Source: Perspect Med Educ. 2022 Mar 17;11(2):93–100. doi: 10.1007/s40037-022-00709-9 (PMC8941044; doi:10.1007/s40037-022-00709-9)
Supplement: Supplementary file 1 — Interview guide for the study: “Reframing professional identity through navigating tensions during residency: A qualitative study” [file 40037_2022_709_MOESM1_ESM.docx]

**Appendix:** Interview guide for the study: “Reframing professional identity through navigating tensions during residency: A qualitative study”

**Opening Questions:**

1) What does it mean to you to be a good doctor?

[Prompt] How did you learn what it means to be a good doctor?

2) When did you first feel like a doctor?

[Prompt] Can you provide examples of experiences that made you “feel like a doctor”?

3) What do you do now that makes you feel like a doctor? What do you do now that makes you feel less like a

doctor?

**Exploring Tensions:**

4) In your current work, what tensions do you face between what you think medicine *should* look like and what medicine *does* look like?

[Prompt] Can you provide a specific example of a tension you faced, and walk me through

how you dealt with it?

[Prompt] Where do these tensions present themselves to you?

[Prompt] When do those tensions present themselves to you?

[Prompt] What emotions do these tensions elicit? How do you express those emotions?

[Prompt] How do you try to resolve those tensions?

5) What tensions do you face between competing values of medicine? One example of what we mean, outside of medicine, would be the tension between values of security and privacy when it comes to security monitoring.

[Prompts] Some examples might be tensions between empathy and objectivity, efficiency and quality,

standardized approach (quality measures) and individualized approach to patient care

6) How have your views on these tensions changed over time?

[Prompt] How are these tensions experienced differently in residency versus medical school?

[Prompt] How are these tensions different than in other professions? What do non-physicians (outsiders) not understand about these tensions?

**Processing Tensions:**

7) Who do you talk to about those tensions? How do you talk about them?

8) Have you seen other residents deal with tensions? How do they deal with tensions?

9) Have you seen faculty members deal with tensions? How do they deal with tensions? How does that affect your own perspective?

10) What effect does the residency learning environment have on how you deal with tensions?

**Link to Professional Identity Formation:**

11) How do the tensions you face affect what it means to be a doctor?

[Prompt] How has dealing with tensions informed your understanding of what it means to be a doctor?

[Prompt] How has discussing tensions with others helped shape your understanding of what it means to

be a doctor?

12) What is the role of role models or mentors in your identity as a doctor?

[Prompt] Positive role models? Negative role models?

[Prompt] Choose one person you view as a role model. How did this person become a role model?

13) What is the role of experience in shaping your identity as a doctor?

14) What effect does your role as a resident have on your identity as a doctor?

**Dealing with Tensions/PIF:**

12) Who or what has been most helping in helping you deal with these tensions? Least helpful?

13) How should the residency program help you deal with these tensions?

[Prompt] What, if any, role should there be in the formal curriculum?

[Prompt] How helpful are residency appointed wellness groups?

[Prompt] What role does faculty/mentor/clinical supervisor play in helping you? What role should they play?

**Closing Questions:**

14) If you were appointed a role in the residency program leadership, what would you do to help residents deal with tensions and identity?

15) What else would be important for me to understand about this topic?
